# On the Prevailing Influenza

**Published:** 1845-02

**Authors:** James Bryan


### *On the Prevailing Influenza.* By JAMES BRYAN, M. D.

The epidemic which has prevailed during the greater part of December and the present month, presents several features of interest to the medical inquirer. The first symptoms of its approach are a slight chill, or the sensation of creeping along the spine, with cold feet and cold hands. At the same time a pain, sometimes very severe, is experienced in the lumbar region, giving the sensation of "slipping or loosening of the joint." Occasionally these symptoms are accompanied or followed by vomiting, and very considerable gastric distress, which continues from an hour or two to twenty-four or thirty hours. The throat at the same time becomes dry and heated, the fauces feel contracted, and a quantity of tenacious mucous is discharged with difficulty. The tonsils, especially where there is a predisposition to it, were enlarged and painful. The pain very frequently extends to the parotid region, and spreads over the occipital and temporal regions; and in one bad case which I saw, the hemicrania was very distressing for two days.

Almost any predisposition in the system is very much excited; pulmonary affections are most of them made worse, particularly in children, numbers of whom have been cut off with symptoms of croup and pulmonary engorgement. One case of chronic epiphora was so much aggravated, that intense inflammation attacked the lachrymal sac, which made it necessary to open it, and antiphlogistic measures were required for its reduction. In that case the eye became involved, and suffered from the violence of the inflammation.

A patient subject to cardiac irritation, was attacked with severe dyspnœa and palpitation, which were relieved only by the free use of the lancet and the tinct. of digitalis. The blood, in this instance, was deeply cupped, and covered with a heavy buffy coat.

One patient during the attack was affected with a slight pleuritis, which was relieved by the ordinary treatment. Great debility, and general prostration of the voluntary powers, almost invariably accompanies the disease. It lasts from two days to two weeks; and the patient not unfrequently, after a mild attack of the disease, finds himself again under its influence, and even to the third or fourth time. The throat, having regained its healthy condition, will suddenly swell up, and the affection previously considered cured will appear again in full force.

The pulse, in most cases, is hard and corded, the respirations difficult, and the tongue is covered by a thick, long white fur. The appetite is variable. In some cases there is none at all; in others it is not materially affected. In most cases the thirst is considerable, the patient demanding continually small draughts of cold water, which is very grateful to the parched throat and deranged stomach. The bowels are most commonly costive, though not always so. The urine in the early stages, is very high colored, but afterwards variable.

*Treatment.*

Antiphlogistics were generally necessary—cathartics and mostly laxatives; the latter in some cases very much improved by the addition of from one-quarter to one grain of calomel. Diaphoretics, expectorants and diuretics, were found useful in the different stages of the disease. Relapses are so frequent that care should be taken to keep the feet warm and dry, avoid long fasting, or much exposure to the damp and cold air which prevails. The winter, so far, (20th of January,) has, with the exception of a few days in December, been a succession of fine mild days, followed by wet rainy weather; the latter prevails at present.

